# On the Necessity of Multidisciplinarity in the Development of at-Home Health Monitoring Platforms for Older Adults: Systematic Review

**DOI:** 10.2196/59458

**Published:** 2025-02-27

**Authors:** Chris Lochhead, Robert B Fisher

**Affiliations:** 1 Advanced Care Research Centre, University of Edinburgh, Old College, South Bridge, Edinburgh, EH8 9YL, United Kingdom, 44 7957617565

**Keywords:** multi-disciplinarity, gait assessment, machine learning, at-home health monitoring, older adults, elderly, artificial intelligence, AI, gait, development, health monitoring, monitoring, systematic review, monitoring system, barriers, caregiver, efficiency, effectiveness

## Abstract

**Background:**

The growth of aging populations globally has increased the demand for new models of care. At-home, computerized health care monitoring is a growing paradigm, which explores the possibility of reducing workloads, lowering the demand for resource-intensive secondary care, and providing more precise and personalized care. Despite the potential societal benefit of autonomous monitoring systems when implemented properly, uptake in health care institutions is slow, and a great volume of research across disciplines encounters similar common barriers to real-world implementation.

**Objective:**

The goal of this systematic review was to construct an evaluation framework that can assess research in terms of how well it addresses already identified barriers to application and then use that framework to analyze the literature across disciplines and identify trends between multidisciplinarity and the likelihood of research being developed robustly.

**Methods:**

This paper introduces a scoring framework for evaluating how well individual pieces of research address key development considerations using 10 identified common barriers to uptake found during meta-review from different disciplines across the domain of health care monitoring. A scoping review is then conducted using this framework to identify the impact that multidisciplinarity involvement has on the effective development of new monitoring technologies. Specifically, we use this framework to measure the relationship between the use of multidisciplinarity in research and the likelihood that a piece of research will be developed in a way that gives it genuine practical application.

**Results:**

We show that viewpoints of multidisciplinarity; namely across computer science and medicine alongside public and patient involvement (PPI) have a significant positive impact in addressing commonly encountered barriers to application research and development according to the evaluation criteria. Using our evaluation metric, multidisciplinary teams score on average 54.3% compared with 35% for teams made up of medical experts and social scientists, and 2.68 for technical-based teams, encompassing computer science and engineering. Also identified is the significant effect that involving either caregivers or end users in the research in a co-design or PPI-based capacity has on the evaluation score (29.3% without any input and between 48.3% and 36.7% for end user or caregiver input respectively, on average).

**Conclusions:**

This review recommends that, to limit the volume of novel research arbitrarily re-encountering the same issues in the limitations of their work and hence improve the efficiency and effectiveness of research, multidisciplinarity should be promoted as a priority to accelerate the rate of advancement in this field and encourage the development of more technology in this domain that can be of tangible societal benefit.

## Introduction

### Background

With the general increase in global life expectancy and the ever-increasing ratio of retirement-age to working-age people, research in the scope of health and social care for older people is greatly increasing. As people live longer and the aging population grows, governments and international organizations are recognizing the need for a paradigm shift in the way we care for our older populations [1]. Current methods of care typically include a social care system to look after the most vulnerable and frail older adults, but as the size of this population increases at a faster rate than the working population, these institutional models of care at the country level become progressively less practical and more costly. In response to this growing crisis, one solution researchers have sought to apply is the use of novel health monitoring technologies to tackle the problem of labor shortages in health care systems and to improve productivity and efficiency in care [2-11]. This can be in direct terms, for example monitoring systems that assist care professionals carrying out their duties within care settings [12] and also indirectly with at-home monitoring devices designed to reduce the occurrence of injuries by, for example, assessing gait [13,14] and predicting fall events [3,15], with the goal of reducing the potential burden on secondary care and ultimately reducing the burden on the care-home sector and keeping people at home, healthy, and independent for longer [9,16,17].

### Research Hypothesis and Goals

The goals of this work can be summarized by the following 2 research questions:

1. Can we construct an evaluation framework for at-home health monitoring research and justify that a positive score in said framework broadly correlates with a higher likelihood of the research being effective in real-world use?
2. Can we identify the trends if they exist, between the method of research (single-discipline, multidiscipline, and the use of PPI or co-design) and the increase or decrease in effectiveness of said research?

In the context of this paper, “effective research” can be defined as research that is more efficient by being less prone to making oversights already identified in the existing literature across disciplines. We assert that research that addresses the already existing issues in the literature (and thereby scoring highly on our proposed metric) leads to more effective research and ultimately leads to products and platforms more likely to be usable in real-life applications (refer to the Evaluation Framework Justification in the Methods section for evidence of this).

To answer the research questions, this work is split into 2 phases, a scoping phase and evaluation phase. In the scoping phase, an overview is provided of the various systematic reviews across disciplines in the field of at-home health care monitoring for older adults. In doing this, we synthesize an evaluation framework based on the consensus and concatenation of these studies to measure the degree to which individual pieces of research encounter similar common problems. By defining this framework, observations can be made about the degree to which these problems are acknowledged by different types of research teams operating with or without multidisciplinarity, which types of issues are likely to be identified by which types of research, and how stakeholder involvement can improve the likelihood of developing an effective product. To justify this evaluation criteria and to address the first research question, examples of existing technologies in commercial use are positively evaluated by this approach to assert the positive relationship between a high evaluation score and the generation of more effective research, considerate to existing identified issues with real-world use (refer to the Methods section). To our knowledge, there is currently no evaluation methodology designed to measure research application effectiveness, based on multidisciplinary metrics.

In the evaluation phase. a review of individual works in the application of computerized at-home health-monitoring systems is then presented, with these works evaluated against the metric developed in the scoping phase, and with the results analyzed to address the second research question (refer to the Results section). For this evaluation phase, a total of 350 papers were found from the IEEE Explore, PubMed, and ArXiv databases, of which 60 papers were ultimately used, with the inclusion constraints being that the papers had to be individual pieces of original research relating either to the development of technology associated with the various applications of at-home older adult health monitoring or a review into the effectiveness of existing at-home health monitoring applications. This is including, but not limited to, development and deployment of disease diagnosis and progression analysis, fall detection and prevention, lifestyle monitoring, vital-sign monitoring, and smart-home systems. Candidate papers must present methods that have the prospect of or are already actively being tested in an at-home environment in whole or in part.

For this evaluation phase, a total of 350 papers were found from the IEEE Explore, PubMed, and ArXiv databases, of which 60 papers were ultimately used, with the inclusion constraints being that the papers had to be individual pieces of original research relating either to the development of technology associated with the various applications of at-home older adult health monitoring or a review into the effectiveness of existing at-home health monitoring applications. This is including but not limited to the development and deployment of disease diagnosis and progression analysis, fall detection and prevention, lifestyle monitoring, vital-sign monitoring, and smart-home systems. Candidate papers must present methods that have the prospect of or are already actively being tested in an at-home environment in whole or in part.

The Methods section contains the scoping phase and provides an overview of the literature in health monitoring technologies, constructing from them a concise evaluation framework in the form of a 10-point checklist criteria for research application effectiveness, based on consensus drawn from these works. The Results section presents the evaluation phase, reviewing individual pieces of research across disciplines using this evaluation framework. The interaction between multidisciplinarity and stakeholder involvement through PPI and co-design and the effectiveness of research are also analyzed here. Finally, the Discussion section summarizes the findings of this research and makes recommendations for how future health monitoring research should be conducted in order to improve the effectiveness of research in this field.

### Research Contributions

The contributions of this paper can be summarized as follows: :

1. A review of the recent literature of at-home health care monitoring. This is achieved by providing a 2-stage review of the literature, with the first (scoping phase) being a meta-review of reviews in the relevant literature, and the second (evaluation phase) being a review of individual works across multiple disciplines in the area of at-home health monitoring.
2. The introduction of a comprehensive evaluation framework for assessing the likely real-world effectiveness of health monitoring application research, based on 10 key issues consistently identified from analysis of 15 literature reviews in the field across disciplines.
3. The identification and demonstration of various links between the manner of research and the effectiveness of research. We find that multidisciplinarity has a consistently positive effect in this respect, reflected by higher scores on the evaluation framework. We also find a convincing increase in scores when PPI and co-design methods are used.

The goal of this research is to concretely illustrate the necessity of multidisciplinarity for the success of at-home medical technology development and deployment and provide a framework for future research to reference when assessing the usefulness of their own applications.

## Methods

### Scoping Phase: Overview of at-Home Health-Monitoring in Older Adults

To identify the most common barriers to uptake in this field, an overview of 12 systematic and other review papers from 2014 onwards was conducted to identify and collate the common issues within some of the common subdisciplines of at-home health care monitoring: encompassing any autonomous monitoring methods used in a domestic context such as camera-based applications, wearable sensors, remote sensors, and ensemble smart-home systems. Table 1 provides a concise summary of the main reviews investigated, and the presence of each of the commonly identified barriers among them. Broken down in Table 1 are the makeup of the teams involved in the research, multidisciplinary, technical, or application-based. In the context of this work, “Technical” concerns research conducted by engineering or computer science teams, with “Application” making up all other types of research team, but predominantly teams in the fields of medicine and social science. In total, 10 core barriers are identified across these reviews and synthesized to be inclusive of all the barriers identified in all the systematic reviews evaluated in this section. An exact breakdown of the presence or absence of the 10 points in each review is available in the supplementary materials (refer Table S1 in Multimedia Appendix 1).

**Table 1.** Overview of systematic reviews and their evaluation score according to the evaluation metric.

Paper title and reference	Description	Research team	Score (%)
Are Active and Assisted Living applications addressing the main acceptance concerns of their beneficiaries? [18]	Overview of opinions of older adults regarding concerns with ambient assisted living technologies.	Multidisciplinary	50
A critical review of smart residential environments for older adults with a focus on pleasurable experience [5]	Review of older adults in focus groups for a variety of smart home applications.	Multidisciplinary	30
Smart homes and home health monitoring technologies for older adults:A systematic review [6]	Investigation of the abilities of various smart-home technologies, alongside feasibility and technical limitations.	Application	70
Health Monitoring Using Smart Home Technologies: Scoping Review [8]	Review of smart home environments, specifically on existing study design limitations alongside technical limitations.	Application	50
Older persons have ambivalent feelings about the use of monitoring technologies [2]	Series of 5 focus groups of older adults attempting to build consensus on the concerns of smart home implementations.	Application	60
Older adults’ perceptions of technologies aimed at falls prevention [3]	Systematic review of focus group-based studies to build consensus on the main factors hindering at home uptake.	Application	40
Unobtrusive sensing and wearable devices for health informatics [11]	Overview of 4 main sensor-based monitoring technologies including relative benefits and drawbacks.	Technical	40
Wearable sensors for remote health monitoring [7]	Overview of specifically wearable health monitoring technologies, mostly centered on technical benefits and limitations regarding data.	Technical	40
Unobtrusive health monitoring in private spaces: The smart home [10]	Overview of smart home. Applications with a focus on perceived “unobtrusive” applications.	Technical	20
Detection and assessment of Parkinson’s disease based on gait analysis:A survey [14]	Overview of gait assessment monitoring for age-related disease detection using a variety of classical and ML^a^-powered monitoring technology.	Technical	0
Remote patient monitoring using AI^b^ [9]	Overview of classical and deep learning-based AI applications in primarily at-home patient monitoring.	Technical	20
Factors Determining the Success and Failure of eHealth Interventions [16]	Overview of smart home. Applications with a focus on discovering the key factors behind the success or failure of med-tech applications.	Multidisciplinary	70

a ML: machine learning.

b AI: artificial intelligence.

“Research Team” denotes whether the teams conducting the research were technical or medical-based. See further in this section for a breakdown of the 10 common identified points and refer to the supplementary material for a specific breakdown of which barriers are present in each. The score value was computed by aggregating which of the 10 features were addressed in each review. Beyond these key reviews from which the evaluation framework was constructed, there are numerous other reviews and surveys whose findings are inclusive of the framework outlined at the end of this section (refer to Table S2 in Multimedia Appendix 1 for a full breakdown of the 13 reviews). Only the core 12 are included here for brevity, with these 12 being selected as they encompass all of the core at-home health care methodologies, include examples of all 10 key points that make up the framework, and represent a roughly equal variety of research teams, namely multidisciplinary teams (n=3), computer science (n=3) engineering (n=2), medical (n=2), and social science (n=2).

**Table 2.** Scoring of 2 current at-home monitoring projects being developed with the intention of widespread use. The factors are numbered corresponding to the attribute list given in the attribute list above. Y or N indicates "yes" or "no" as to the presence of the factors.

ProjectName	Description	Factors (Y or N)									
		1	2	3	4	5	6	7	8	9	10
SPHERE^a^[19-21]	Smart-home system for behavior monitoring, under development for several years and the feature of multiple research studies.	Y^b^	Y	Y	Y	Y	Y	Y	Y	N^c^	Y
HALLEY^d^[22]	Internet of things-based Smart-home development project currently in commercial development.	Y	Y	Y	N	Y	Y	Y	N	N	Y

a SPHERE is a multi-year smart-home development project based in England which has been involved both in commercial smart-home production as well as research.

b Y: yes.

c N: no.

d HALLEYASSIST is a company based in Australia involved in developing multiple healthcare monitoring devices for a smart home context.

The study by Ghorayeb et al [23] presents a number of insights in their review of the literature regarding older adults using smart-homes, such as identifying solutions to the problems in most research applications. For example, they assert that gradual introduction of smart-home technology combined with the ability to “pause” it at will to provide “emotional release” is highly desired in end users for a more pleasant experience. They also find that more tech-literate people tend to have less concerns with the technology due to an improved understanding of the data being collected and the privacy risks involved, if any. One other concern identified was transmission of data and the insecurities associated with this, leaning to a user-preference that data should be handled manually instead of through remote connection. These findings are all further reinforced by the findings in [24], which similarly conduct interview sessions with 20 older adults regarding which aspects of health-monitoring systems concern them. These findings inform points 4 and 5 in the evaluation metric, defined in Methods section.

Regarding data access, the study by Robinson et al [25] finds that older adults could in some cases want control of data access to withhold the data from certain parties, namely their family and friends, for fear of burdening them as well as the parallel desire some express that they do not want to be micromanaged by their loved ones or health care providers, informing points 6 and 8 of the framework. Cost was also an identified challenge (informing point 3), for example focus group attendees in [2] were concerned by the cost of long-term use of monitoring devices, with the implication being that they would have to buy them outright.

The study by Hawley-Hague et al [3] discovers that when surveyed, there is a broad perception that older people view new advancements in smart-home related technology as good but “unnecessary for them,” as they don’t perceive themselves as being “unhealthy enough” to merit using what they see as a drastic action toward greater care. Mann et al [26] found in their study of 661 older people that a slim majority (56.3%) perceived smart-home technology as not being of use to them specifically. Ghorayeb et al [23] identify a series of factors through their investigation with older participants which are crucial to account for when considering the integration of new technology into real life. These factors range from societal stigma to technical reliability, covering points 1, 2, 3, and 6 in the evaluation framework. Research in studies by Boström et al and by Boström et al [2] and Liu et al [6] also point to the issues surrounding the implication of autonomous monitoring being a decline in human contact, addressed by point 7. In the systematic review conducted by Ghorayeb et al [23], observations were made regarding the progression of smart-home technologies. Regarding patient acceptability, they note that less than half of the papers reviewed take into consideration the acceptability of their technology where privacy is concerned: with the focus of the paper instead being explicitly about the functions of the novel technology. Some steps taken toward improving privacy in certain papers included encrypting collected data [27] and locking data access behind authorization [28].

Regarding health care professionals, their main concerns with smart-home technology and health care monitoring concern the feasibility of use. Unlike patient acceptability, acceptability by health care professionals is underresearched, where many papers will either focus on the technology being developed and not address it in application or they will focus only on the opinions of the end users and how they will use and accept the technology [29]. This finding is echoed in the lack of surveys found in the scoping review in the Results section, where the focus is on health care professionals rather than patients. Of the 60 papers in the scoping review, only 7 made any explicit use of external medical caregivers in their research development.

### Evaluation Framework Criteria

Across the review papers both in Table 1 and the other reviews referenced in this section, the following list of 10 factors were collated to encompass all the common considerations identified when designing and implementing novel monitoring technology for use with older adults in a domestic environment. They are segmented by category of concern (technical, application, or multidisciplinary), where the technical are purely engineering or computer-science implementation concerns and application are concerns involving human interaction with the technology and any adjacent concerns important to medical and social science-based disciplines. The framework is as follows (Textbox 1).

Textbox 1. **The evaluation framework**

- Usability-technical: concerning the use of the technology and how feasible it is to be used by caregivers, health care professionals, and end users.
- Accessibility-technical: this concerns the ease of use by laypeople of the computer technology and the barriers for entry in terms of effectively using the technology.
- Reliability-technical: covers issues relating to the long-term viability of the application, for example, is it expensive, does it require upkeep, charging or maintenance.
- Control-multidisciplinary: concerns the level of access both patients and health care staff should have to the application and the data it records.
- Privacy-multidisciplinary: constitutes issues relating to the intrusiveness of the data being collected, and the manner in which it is collected.
- Stigma-application: anything relating to the societal pressures and negative connotations people may feel by using monitoring technologies for the purposes of personal health.
- Lack of human response-application: concerns the issue of the perception that increased autonomous monitoring would result in a decrease in person-to-person interaction as a result.
- Burden to others-application: regarding the perception of older adults that additional at-home monitoring is representative of applying additional pressure on their caregivers.
- Lack of perceived need-application: concerns a commonly identified phenomenon in the literature that people have a tendency to think a technology is useful but unnecessary for them personally.
- Affordability-application: this concerns the cost both on a personal and institutional level to implement solution applications in real life.

### Evaluation Framework Justification

To demonstrate the descriptive power this evaluation system has to the likelihood of success in application, Table 2 provides an overview of papers concerning 2 technologies being developed for commercial use (Sphere [21] and HalleyAssist [22]), and the degree to which the prior research and development of these systems adhere to the criteria in the evaluation metric, with scores being calculated based on all existing literature regarding each technology, as opposed to individual articles. Both have demonstrated a commitment to addressing barriers relating to both technical and human elements, addressing 90% and 70% of the evaluation metrics respectively (the average score across the scoping review in the Results section is 36.2% for comparison). They also both were developed by a multidisciplinary research team from computer science and signal processing to nursing, geriatric medicine and social care, with both systems now being in varying stages of commercialization. The relationship between the absence of certain metrics such as multidisciplinarity and the lack of consideration for common barriers to uptake is further concretely illustrated during the analysis at the beginning of the Results section. While these are only 2 data points and cannot be said to constitute a trend on their own, it can be asserted that they provide a strong positive indication that successful applications are likely to score highly on the introduced metric.

## Results

### Evaluation Phase: Scoping Review and Parameters

**Figure 1.**
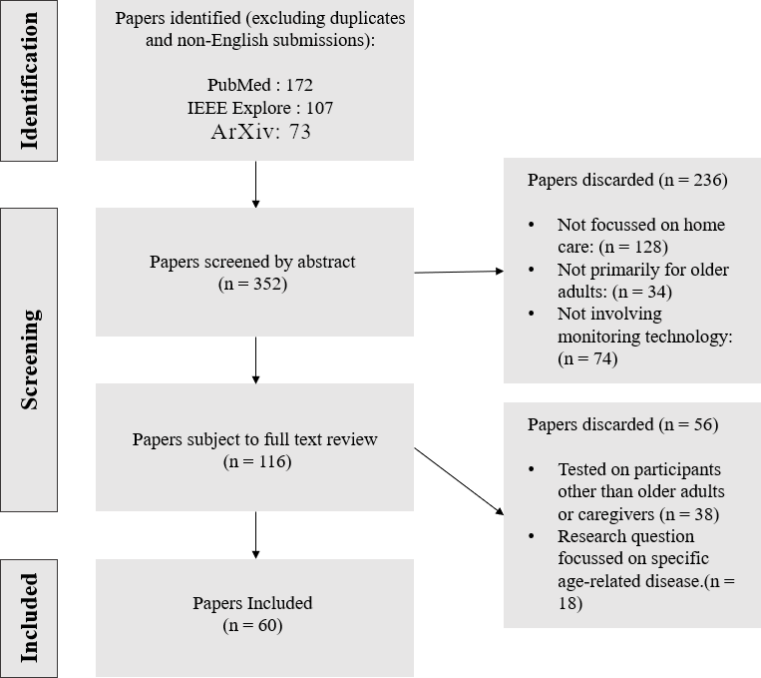
PRISMA (Preferred Reporting Items for Systematic Reviews and Meta-Analyses) flow diagram: screening process illustration for the scoping review portion of this research.

Figure 1 illustrates the selection process for papers considered for the scoping review. From the 3 databases, the following search term was used:

((healthcare monitoring) AND (older adult)) AND ((home) OR

(at-home) OR (domestic)) AND ((obtrusive) OR (unobtrusive) OR (intrusive)) AND ((machine learning) OR (AI) OR (artificial intelligence))

with the exception of the ArXiv database, where the

((obtrusive) OR (unobtrusive) OR (intrusive))

section was omitted for a larger range of initial texts. Language was restricted to English and the date was restricted to 2014 or later, with the search commencing on January 7-24, 2024. Excluded were papers that did not in whole or in part reference older adult monitoring as the purpose of their application or review, and papers where the application was specifically designed for care homes or hospitals. The initial search yielded a total of 352 papers. Paper title and abstract analysis excluded 236 papers, which resulted in 116 remaining, with the final count being 60 papers after a full-paper review resulting in 56 more deletions (Figure 1).

The following section is broken down by technical and application-based research, following the definitions for both established in the beginning of the Methods section. Papers defined as multidisciplinary are those in which the research team consist of at least 1 person from each category.

### Technical Research

The majority of research in this space relies either on traditional or neural network–based machine learning methods to make their systems effective and largely automatic. Researchers in [30] construct a machine learning model that can differentiate between regular, prefrail, and frail gait within a population of 50 older adults at an accuracy of 88.5% based on gait data collected from wearable accelerometer signals. They rely mostly on “traditional” methods of machine learning: that is, machine learning methods that don’t use deep neural networks.

With vision-based applications, on the other hand, deep neural networks, especially convolutional networks designed specifically for processing image data, are frequently used. Researchers in the study by Lin et al [31] use a novel 3D convolutional neural network (CNN) to process gait silhouette images from the CASIA-B dataset [32] to achieve a person-identification accuracy of 97.6%. While the question of person identification through gait can be fairly trivially solved with modern CNN frameworks, the question of gait analysis, or assessing health requires a more nuanced approach and is still an active research area.

Researchers in [33] use a Spatiotemporal Graph Convolutional Network (ST-GCN) [34] framework to assess Parkinson disease severity as classified under the Unified Parkinson’s Disease Rating Scale [35]. The ST-GCN is an extension of the standard CNN model that works specifically on skeletal graph data as opposed to raw images, and works by processing input data with sequential attention to both the spatial and temporal dimensions of the input. As gait assessment is a far more complicated problem than simple person-identification or even traditional action recognition, they achieved 53% *F*_1_-score using a dataset of 53 individuals with Parkinson disease.

Recently, in the study by Yin et al [36], the Spatio-temporal joint adjacency GCN (STJA-GCN) was developed that uses 3 input streams for joints, bones and velocities, a novel joint attention module to emphasize spatial data and a simplified skeletal graph input architecture to achieve state-of-the-art results (93.17% and 92.08%) on the domain of recognizing and classifying different types of abnormal gaits compared to other ST-GCN–based methodologies. This was tested on a pair of synthetic gait datasets introduced by the researchers, totalling 22 participants and 8600 instances of gait data collected from a total of 7 sensors across the 2 datasets.

**Figure 2.**
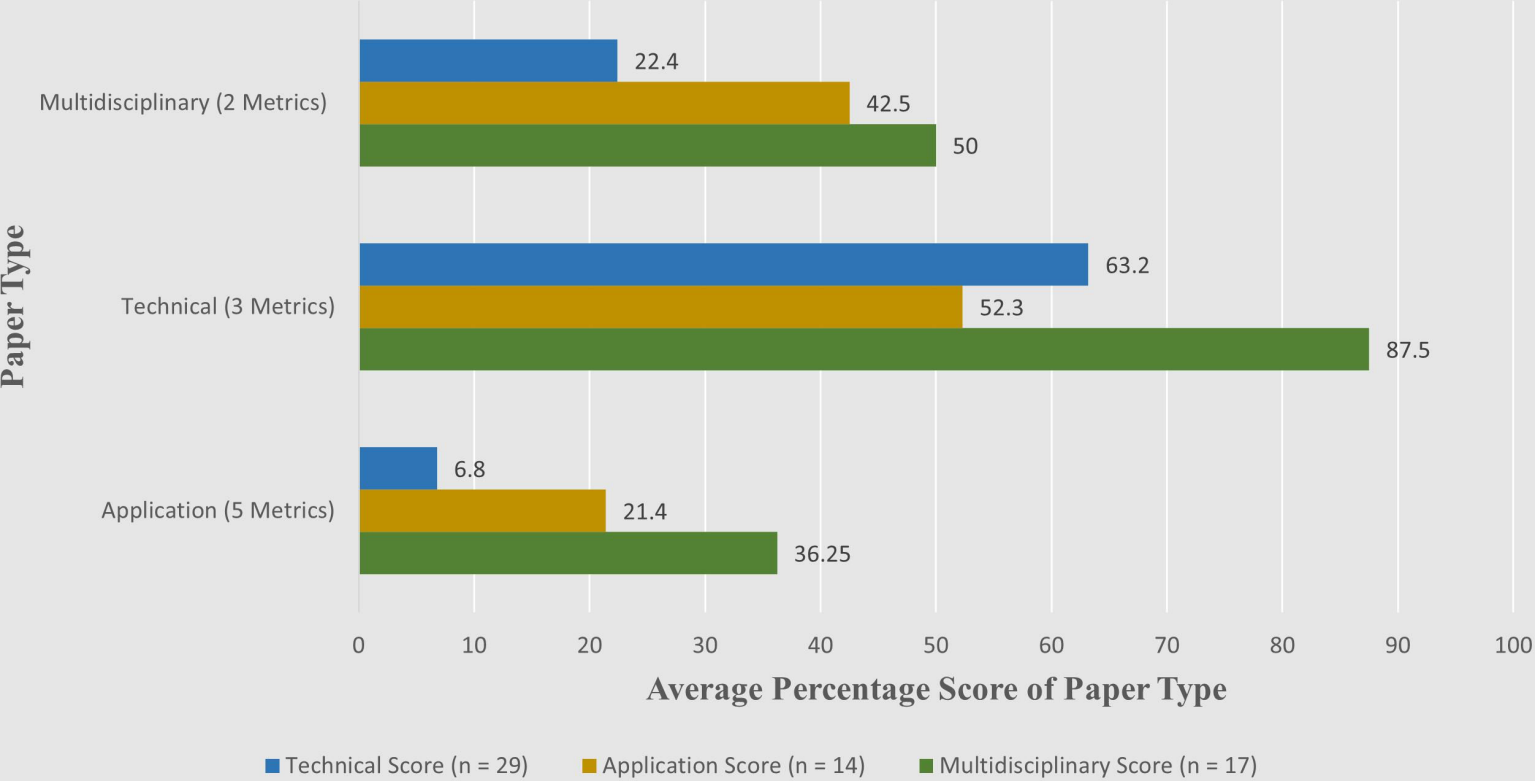
Illustration of evaluation performance per-evaluation category. Shown here is the impact that the use of multidisciplinarity has on the likelihood of each of the 10 metrics being taken into consideration.

**Figure 3.**
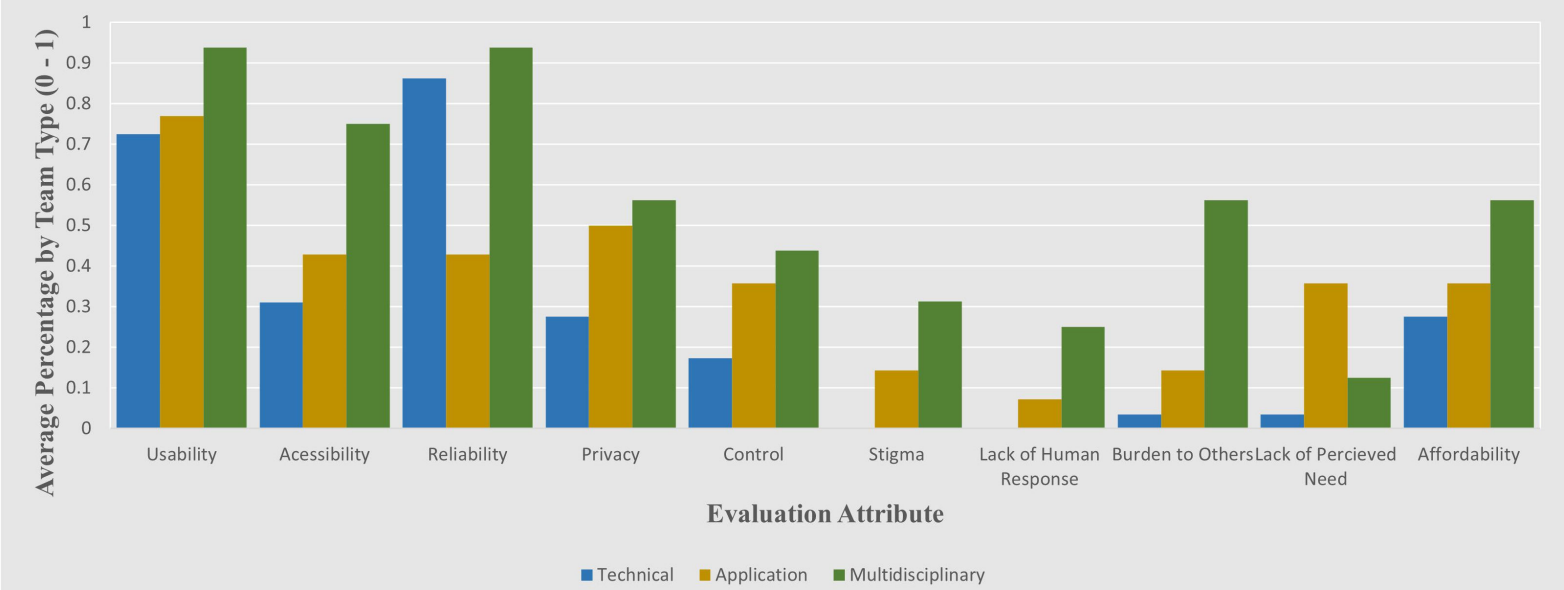
Breakdown of the average presence of each evaluation attribute by research team type.

Demonstrably, machine learning methods are extremely capable in multiple health care monitoring applications, necessitating the inclusion of highly technical fields in this type of research. The issue with the papers in this field, however, is that they score poorly on the proposed evaluation framework due to an overt focus on technical innovation at the expense of applicational concerns as well as scoring consistently lower than papers in the applicational and multidisciplinary categories on all but explicitly technical metrics (Figure 2). This analysis indicates a narrow focus almost purely on the effectiveness of the technology at the expense of an omission of application considerations, such as cost or perceived need by end users and medical stakeholders (refer to Figure 3 for a per-category breakdown). End users in this context refers both to patients and health care professionals who would be involved with the use of the technology (such as general practitioners, nurses, physiotherapists, occupational therapy and care workers). Stakeholders, on the other hand, refers to family members, friends or dependents of the patient using the technology. To take a more concrete example of this narrowed focus, research by Brunzini et al [13] is explicitly geared toward the development of a decision-support algorithm for health care professionals, complete with bespoke visual aids. However, there is no evidence of consideration of the requirements of health care professionals who would prospectively use such a system (in this case namely GPs, geriatricians, and nurses) being used in the design of the system itself. Similar issues were found in several studies where technical teams sought to develop assistive tools for caregivers or patients without direct involvement of prospective caregivers or end users, ranging from tools designed for doctors and nurses to carers, physiotherapists, and occupational therapists [37-41].

### Application and Multidisciplinary Research

Of the surveyed technical papers (n=29), they scored an average 6.8% on the application-centered metrics in the proposed evaluation framework (Figure 2). In contrast, the application papers (n=14) mainly authored by health care researchers scored 21.4% on application-based metrics. While this indicates that application focused papers typically address more concerns with real-life deployment of research solutions, there is also a notable trend that this focus on application comes at a slightly increased tendency to neglect technical aspects of research (with both technical and multidisciplinary papers outperforming application-based papers on technical metrics). A clear trend can be seen in multidisciplinary papers consistently outperforming technical or application-based papers, even on the metrics specific to their own discipline (Figure 3).

As indicated in the second research question in the Introduction, we speculate that there exists a relationship between the inclusion of different stakeholders in PPI and co-design with the effectiveness of the resultant applications and research. As indicated by Table 1, this type of inclusion is usually implemented using focus groups. In this section, we highlight the typically moderate-to-high scores where this stakeholder input is prioritised, whilst also outlining other areas of shortcomings that follow from lack of multidisciplinarity in other, mainly technical domains.

In the study by Mireles et al [42], a total of 75 health care professionals (mostly nurses, n=47) are interviewed as a means of investigating the effectiveness of 3 different eHealth technologies for at-home monitoring of older adults with chronic conditions. Scoring 40%, this research focuses on the implications of e-health solutions as they affect end users, namely stakeholders, with less consideration for the technical implications of their recommendations. Research in the study by Bjornsdottir and Ceci [43] is similar, conducting qualitative interviews with both health care professionals and also patients. By including the patients and getting their insight, this research scores higher (60%) but suffers on the same technical metrics, highlighting the shortcomings in the systems they are evaluating without explicit consideration of the technical implications of their conclusions. In [44], 5 focus groups were conducted with both older adults and caregivers; namely family and care staff, to help synthesize a consensus on the design of future at-home sensor-based systems, with a focus on acceptability, respect for privacy and how to best provide control of care to the end users themselves. Scoring 50%, the authors neglect aspects such as system reliability at the expense of greater privacy, and other application-driven barriers such as societal stigma and the threat of greater isolation resulting in the deployment of remote monitoring technology. Expanding on this focus-group centered research [45], conduct a series of interviews with both formal and informal caregivers to identify the key parameters which need to be addressed to achieve effective at-home lifestyle monitoring systems. Scoring 60%, their strengths come from the inclusion of both medical and end user input, however they similarly lack extensive consideration for how the parameters being set by stakeholders would affect the performance of the monitoring systems themselves. Itoh et al [46] investigated issues of application from a medical perspective but score only 40%. Not only does their investigation consist only of testing an existing technology without the intention of developing said technology further, they also concern their research only with application concerns as far as the caregivers and medical stakeholders are concerned, with no involvement being afforded to the end users and patients. As a result, issues around stigma and even privacy are completely unaddressed.

As can be seen in Table 3, there is a clear lack of involvement in research and application design from the medical community and caregivers. Overall, 13.7% and 64.2% of single-discipline papers involve end user participation versus a mere 14.2% and 0% exhibiting caregiver or medical participation in the development of the research (for application and technical based papers. respectively). While the likelihood of involving both types of stakeholders (caregivers and end users) is more than double when multidisciplinary teams are involved (14.2% vs 37.5%), this is not conclusive due to the relatively small number of papers in each category when divided by the inclusion of different stakeholder types (end user and caregiver). Likewise, there is a definite trend across all research team types that exhibit lower evaluation scores when neither caregivers nor end users participate in the design or evaluation of research. However, discerning the differing impact between the 2 stakeholder types is difficult due to the small positive sample size (with only 2% separating end user only and caregiver only scores for both research team types, refer to Table 4). The trend in the research, especially in survey-style research seems to be to focus on end users rather than caregivers or other medical stakeholders with only 7 of 60 papers involving medical stakeholders and 22 of 60 involving end user input.

**Table 3.** The presence or absence of caregivers and end users in the co-design of research across the review, split by research team type.

Team type	Caregivers (%)	End users (%)	Total (%)
Multidisciplinary^a^	37.5	56.25	*37.5*
Application^a^	38.4	64.2	14.2
Technical^a^	0	13.7	0

a Values are not mutually exclusive and some papers have both caregivers and end users involved. As a result, values may not necessarily add up to 100%.

**Table 4.** The average evaluation score by research team type when caregivers and end users are included or not in the research.

Team type	Neither (score %)	Caregivers (score %)	End users (score %)	Caregiver and End users (score %)
Multidisciplinary	35.7	70	68	70
Application	27.5	40	42	43
Technical	24.8	0	35	0

The strongest scores using the evaluation framework come from those where interdisciplinary teams are used (average score of 5.43 versus 3.5 and 2.68 for application-based and technical teams respectively), with a distinct trend shown in Table 4 indicating the presence of caregiver and end user involvement in a co-design or PPI capacity leads to a stronger evaluation score.

In general, as shown in Figure 3, different categories of research team tend to neglect certain aspects identified in the evaluation framework. Technical papers struggle with “Stigma” and “Human Response,” and application-based papers, mostly led by medical teams often omit factors such as “Accessibility“ and “Burden to Others” from their consideration. Both tend to struggle particularly with addressing societal stigma, possibly due to the varying levels and types of stigma for at-home monitoring from researcher’s home countries, or potentially due to considerations for more general issues not directly related to their specific research being deemed as outside of the scope of their work.

A common theme across all of these works is the tendency to make assumptions on the necessity of their application in the eyes of end users (Figure 3), as the lack of perceived need is seldom addressed regardless of the make-up of the research team. Technical issues are generally well addressed such as usability and reliability, however broader human issues such as societal stigma and feelings of end users becoming a burden, or the technology itself being a burden to use by caregivers are likewise rarely addressed. Across the entire evaluation phase, there is a consistent trend of greater representation of every attribute when multidisciplinary teams are involved in the research, with specifically technical teams being especially susceptible to a lack of focus on application-based concerns.

## Discussion

### Principal Findings

The research presented here concludes that a root problem underlying the issues affecting real-life uptake of research applications is the lack of multidisciplinarity in the research. We find that the developed evaluation metric is effective for quantifying the likelihood an application will transition into further development and effective deployment in real-world use. We also identified a series of common themes across the literature, namely the consistent underrepresentation of end users and other stakeholders in research, a distinct lack of multidisciplinarity in technical applications and the consistent underappreciation of societal factors such as stigma and human contact when developing at-home medical technologies.

Using a novel evaluation metric based on the 10 key barriers to uptake identified in the literature, a concrete trend can be observed that that multidisciplinarity between engineers, computer scientists, medical experts, and social scientists, alongside co-design or PPI-based inclusion of caregivers (such as family, friends, or dependants) and end users (health care professionals or patients) is highly beneficial to the effective development of technology that has direct practical application and tangible social benefit. The evaluation framework itself was also justified by the demonstration of a trend of extremely high evaluation scores on research projects that had reached the point of successful practical application, namely HalleyAssist and SPHERE. Using this evaluation metric, we find an increase of between 19.3%‐27.5% for multidisciplinary teams versus single discipline, and an increase of between 7.4%‐19% when research includes the use of caregivers or end users in a PPI or co-design capacity.

### Limitations and Future Work

The main limitation of this study is that the results can only demonstrate the lack of multidisciplinarity is a root issue, not necessarily the root issue. For example, many extenuating factors not included in the research itself can explain why effective technology was researched but not developed, such as personal material conditions for the researchers, leading them to be uninterested or unable to develop effective research further. One other limitation is in the methodology of the framework itself. Ways to improve the descriptive power and nuance of the framework could be to introduce more criteria, break the criteria down into further subdisciplines or introduce criteria weighting so that criteria deemed more “vital” or less common are weighted higher when calculating the score. Had more resources been available, a broader review in both phases could have been conducted, using a greater number of papers from a greater number of databases, an especially important factor given the number of different disciplines relevant to this type of research.

In future, a larger study could be conducted on the literature to further verify the effectiveness of the evaluation framework, including the application of the framework to more smart-home technologies in commercial development. The framework itself could also benefit from being imbued with greater complexity for example, additional factors or a bespoke weighting of them to make certain factors more important to the success of a technology in application, and thus scoring a higher score. To our knowledge there is no comparable work in quantitatively assessing the effects of multidisciplinarity on research in at-home health monitoring, so in general, more research is needed to saturate the domain and allow for trends to be more concretely identified. As HalleyAssist and SPHERE are leading examples in the area of in-home monitoring of ageing singles, it would be good to follow up their development and experiences in the future, to see how well the assessment undertaken here was predictive of their successes.

## Supplementary material

10.2196/59458 Multimedia Appendix 1 Supplementary materials referred to in the manuscript. There is also a live hyperlink.

10.2196/59458 Checklist 1 PRISMA checklist.

## Abbreviations

CNN: convolutional neural network
PPI: public and participant involvement
ST-GCN: Spatiotemporal Graph Neural Network
STJA-GCN: Spatio-temporal Joint Adjacency Graph Neural Network
